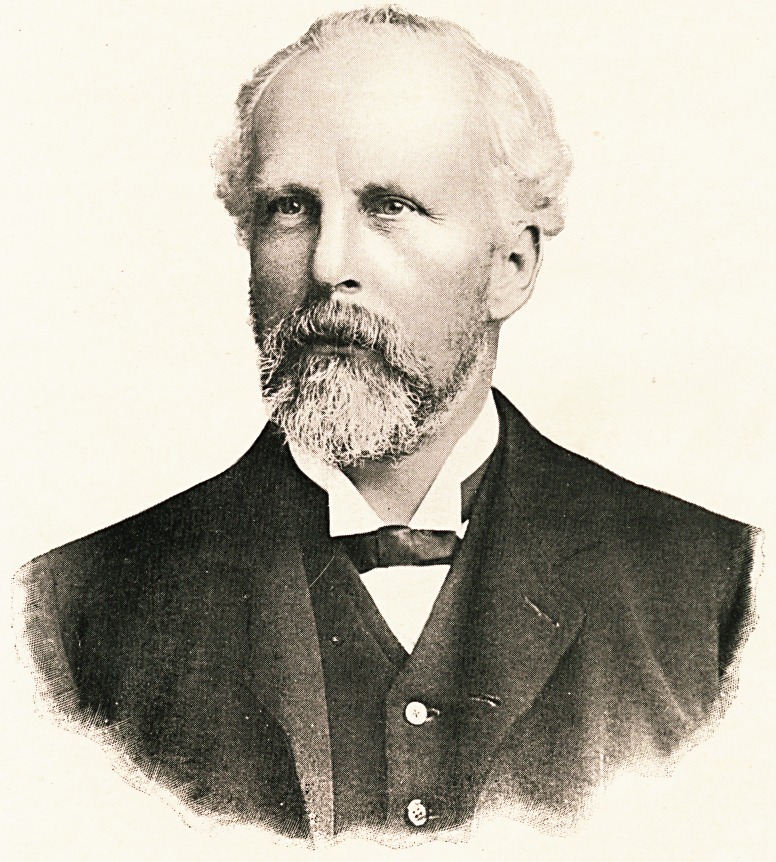# Alfred Edward Aust Lawrence, M.D., C.M.

**Published:** 1901-09

**Authors:** 


### The Bristol

### Medico-Chirurgical Journal.

SEPTEMBER, 1901.

### ALFRED EDWARD AUST LAWRENCE, M.D., C.M.

Dr. Lawrence's many friends, both medical and lay, heard with deep regret of his sudden and quite unexpected death, which took place on August 29th at Bishop's Teignton, in Devonshire, where he was spending part of his summer holiday.

On the day previous to his death he was on the moor; and afterwards dined with his wife and daughter, passing the remainder of the evening with the guests staying in the house, apparently in the best of health and spirits. His bedroom was near to that occupied by his wife and young daughter. In the morning Mrs. Lawrence, on reaching the breakfast-room, found that her husband had not yet come down; she therefore went up to his room, and found him in what she thought was a faint. Dr. Sargent, however, who was staying in the house, found life to be extinct, the appearances being such as to lead to the supposition that he had died in his sleep. After an autopsy, Dr. Wood, of Teignmouth, stated at the inquest that in his opinion death was due to syncope from heart failure.

Dr. Lawrence was born in Bristol in March, 1848, and was, therefore, in his 54th year. His father was a well-known Bristol citizen; and there were numerous brothers and sisters.

Dr. Lawrence was educated at the Bristol Grammar School, and had for schoolfellows some who are still practising in this city. He was a student at the Bristol Medical School and General Hospital. At this latter place, it was then customary for a student to enter under a particular surgeon, and young Lawrence became the pupil of Mr. Coë; he obtained many prizes, but held no resident appointment as he was anxious to go to Aberdeen University, where he took the M.B., C.M. in 1872, and the M.D. degree in 1874. He married, and started in general practice in Clifton in 1872. In 1875 he was appointed Physician - Accoucheur to the Bristol General Hospital, in succession to Dr. J. G. Swayne; this post he held for twenty-one years, on retiring from which he was appointed Consulting Physician - Accoucheur, in appreciation of conspicuous services to the Hospital. At the time of his election, the duties of the office were mostly confined to the out-patients, and no beds were allotted to him as a right, nor was he allowed to perform abdominal operations; but Dr. Lawrence was chiefly instrumental in establishing the well-equipped Women's Department which now exists at the Hospital. This was probably the great work of his life: placing, as it did, himself, his successors in office, and the department, in a well-defined and satisfactory position.

At the time of his death Dr. Lawrence was Professor of Midwifery in University College, Bristol; and he had formerly been Demonstrator of Anatomy in the Medical School. He was also Vice-President of the Obstetrical Society of London. In 1896 Dr. Lawrence was President of the Bristol Medico-Chirurgical Society; his Presidential address being on "The Evils of Marriage and Pregnancy in Women who do not possess Sound Pelvic Organs." This address was published in this *Journal* (Vol. xiv., 289), to which he contributed many papers in connection with his special subject. He was a regular attendant at the meetings of the Society, and took a warm interest in its welfare. Dr. Lawrence was President-Elect of the Bath and Bristol Branch of the British Medical Association; and was, as the writer of this notice knows, looking forward to his year of office with much interest and pleasure. This is the first instance for the last thirty years in which a President or President-Elect of the Branch has died during his year of office. Dr. Lawrence was in full practice up to the time of his death, and for many years had confined himself almost entirely to special work. His advice and assistance were constantly sought by his professional brethren, both in Bristol and in distant parts. As a consultant he was careful and thorough in his examination of the patient, prudent and cautious in forming an opinion, but firm and decided in giving expression to that opinion when once formed; inclined to be rather over-anxious as to the welfare of those under his care, he was at the same time calm and resourceful in difficulties; he was manly and straightforward in all his dealings both with patient and practitioner; and his loss will be long felt by the profession, as well as by the public.

Dr. Lawrence was very fond of horses; in the winter he generally put in the best part of one or two days a week with the Badminton or Berkeley hounds, and contrived to get a considerable amount of recreation and enjoyment out of this, his favourite pastime. In manner Dr. Lawrence was always bright, cheery, and pleasant; he never nursed a grievance: if he had one, he fearlessly faced it; he had it out, and there was an end of it. In person he was of medium height, and somewhat strongly built; but of late years his clear and rather ruddy complexion contrasted sharply with the prematurely white and wavy locks which were so becoming to him, but which gave him a more venerable appearance than was justified by his actual tale of years.

He leaves a widow, two daughters, and many friends to mourn his comparatively early death. He was buried at Redland Green, not far from the graves of Augustin Prichard, Henry Marshall, and Greig Smith—a goodly company.

At the graveside there was a very large attendance of the medical profession and others; notwithstanding the fact that this is the period of the year when many are still from home, there were few practising in Bristol and the neighbourhood who were not present: a striking testimony to Dr. Lawrence's popularity—a popularity not gained by the influence of social amenities, but due entirely to professional ability and personal integrity.

The *Journal* Committee wish to direct especial attention to the paper by Dr. Aust Lawrence in this *Journal* on "Some of the Hemorrhages of Pregnancy," and regret that it is the last opportunity they will have of publishing the results of his matured experience and well-balanced judgment.

## Figures and Tables

**Figure f1:**